# Case Report: Atypical presentation of rickets with hypocalcemia-related emesis

**DOI:** 10.3389/fped.2025.1627190

**Published:** 2025-07-31

**Authors:** Andrea Francioni, Verena Simone, Elisa Laschi, Luisa Lonoce, Francesca Mugnai, Michele Minerva, Davide Cherubini, Salvatore Grosso

**Affiliations:** Clinical Pediatrics, Department of Molecular Medicine and Development, University of Siena, Azienda Ospedaliero-Universitaria Senese, Siena, Italy

**Keywords:** rickets, vitamin D, hypocalcemia, emesis, vomit, infant, case report

## Abstract

**Background:**

Nutritional rickets, primarily resulting from vitamin D and/or calcium deficiency, is a well-recognized cause of skeletal and extraskeletal manifestations in children. However, gastrointestinal (GI) symptoms, such as vomiting, are not commonly reported as primary manifestations associated with hypocalcemia at the onset.

**Case presentation:**

We describe a case of a 9-month-old male infant of Afghan origin who presented to the Pediatric Emergency Department with a 7-day history of isolated postprandial vomiting. Physical examination revealed a large anterior fontanel, but no significant skeletal abnormalities. Laboratory blood evaluation demonstrated severe hypocalcemia, mild hypokalemia, and elevated alkaline phosphatase. Arterial blood gas analysis confirmed low ionized calcium and revealed metabolic alkalosis. Electrocardiogram showed a prolonged corrected QT interval (QTc). Intravenous administration of calcium gluconate and potassium led to rapid normalization of electrolytes and resolution of vomiting and QTc prolongation. Further investigation revealed severe vitamin D deficiency and elevated parathyroid hormone, consistent with nutritional rickets, which was confirmed by wrist radiographs. Oral supplementation with vitamin D3 and calcium carbonate resulted in complete resolution of symptoms and biochemical normalization at discharge and follow-up.

**Conclusion:**

Hypocalcemia can contribute to GI dysmotility and altered gastric secretion thus vomiting may be a possible symptom related to electrolyte disbalance of rickets. To date it is the first case report reporting isolated postprandial emesis as a presenting symptom of nutritional rickets. Clinicians should be aware of this unusual presentation to facilitate timely diagnosis and appropriate management, particularly in at-risk populations.

## Background

Rickets is a heterogeneous group of congenital and acquired disorders characterized by altered calcium and/or phosphorus homeostasis with concomitant disturbance of mineralization of the growing skeleton (1). The most common cause of rickets is reduced nutritional intake of calcium and vitamin D, though the development of the latest diagnostic techniques has increased the identification of different genetic forms of rickets (2).

Nutritional rickets is characterized by a defective chondrocyte differentiation and growth plate mineralization along with altered osteoid mineralization and it is caused by vitamin D deficiency and/or low calcium intake in children (3). It was widespread over 200 years ago in developed countries, with a reported prevalence of 25% among children in the United States at the beginning of XIX century (4). Subsequently, the introduction of vitamin D fortification in formula milk during the 1950s has dramatically reduced the incidence of nutritional rickets, but this disorder has been still being reported nowadays with a global estimated incidence of 2.9–27 per 100,000 individuals (5), especially in dark-skinned infants who are breastfed after 6 months of age. Indeed, breastfed children are particularly prone to rickets because of the high prevalence of vitamin D deficiency in mothers associated with the low content of vitamin D and its metabolites in breast milk. Furthermore, these children may experience lack of exposure to sunlight and increased use of sunscreen and other skin-protective barriers. Globally, nutritional rickets seems to be more common in the Indian subcontinent, Africa and the Middle East but it is still reported even in developed countries, where the prevalence is higher among immigrant individuals, those with dark skin and those with minimal sunshine exposure. Overall, rickets still remains a major public health problem and a preventable cause of marked morbidity and mortality (4, 5).

The clinical presentation is heterogeneous, including skeletal and non-skeletal manifestations, and it depends on the age of onset and the predominant pathogenetic mechanisms. The typical clinical features are skeletal deformities particularly of the lower limbs, softening of skull bones (craniotabes), frontal prominence of the skull, growth retardation, delayed tooth eruption, enlarged fontanelles, short stature and joint alterations (6). Severe forms of rickets may present with the so-called “rachitic rosary” (due to expansion of the costochondral junctions) and increased tendency to bone fractures (2, 4, 5). Bone deformities frequently occur during the first year of life and are considered highly indicative of rickets (2).

Extraskeletal manifestations may include muscle weakness, hypotonia, delayed motor development, irritability, lethargy, hypocalcemic seizures, tetany, laryngospasm, dilated cardiomyopathy, anemia, predisposition to respiratory infections (2, 5).

The diagnostic approach to rickets is based primarily on history, laboratory and radiological examinations, followed, if indicated, by genetic testing (1). Nutritional rickets could be diagnosed on the basis of history, physical examination, laboratory tests, and radiographic confirmation (3). However, genetic analysis is recommended in patients with strong suspicion of genetic form of rickets, even in absence of a family history of the disease (2).

Noticeably, prevention of rickets is possible only for the nutritional form, i.e., through dietary supplementation with vitamin D and calcium, alone or in combination with exposure to sunlight.

The primary goal of treatment is to correct or at least improve the pathological picture based on clinical and biochemical parameters. Clinical evaluation includes monitoring of auxological data (height, weight, growth velocity and head circumference in newborns), the degree of deformity of the lower limbs, the gait, the presence of bone pain, muscle weakness and dental abnormalities (7).

Being a relatively common and treatable disease, clinicians should consider this diagnosis in patients presenting with typical or atypical features and belonging to the “at risk” population.

## Case presentation

A 9-month-old male of Afghan origin presented to the Pediatric Emergency Department for a 7-day history of postprandial gastric emesis (maximum 7–8 episodes per day); all the episodes were temporally related to feeding. He had normal bowel movements and no other symptoms. The absence of concomitant gastrointestinal (GI) or infectious symptoms in other family members was reported.

The child was born from non-consanguineous parents and had five other healthy siblings. His perinatal history was unremarkable. An appropriate age achievement of neuromotor milestones was reported. He was exclusively breastfed until six months of age, then he began weaning with good tolerance; he had not been supplemented with vitamin D over the last two months. The parents reported that the child used to spend time indoors, with consequent poor exposure to the sunlight.

On physical examination, he presented good general clinical conditions, good hydration status, normal cardiac, thoracic and abdominal physical examination, large anterior fontanel (2 cm × 3 cm), weight of 10 kg (90th percentile), length of 78 cm (>95th centile) and head circumference of 47 cm (95th percentile); no significant skeletal alterations were observed.

**Figure 1:**
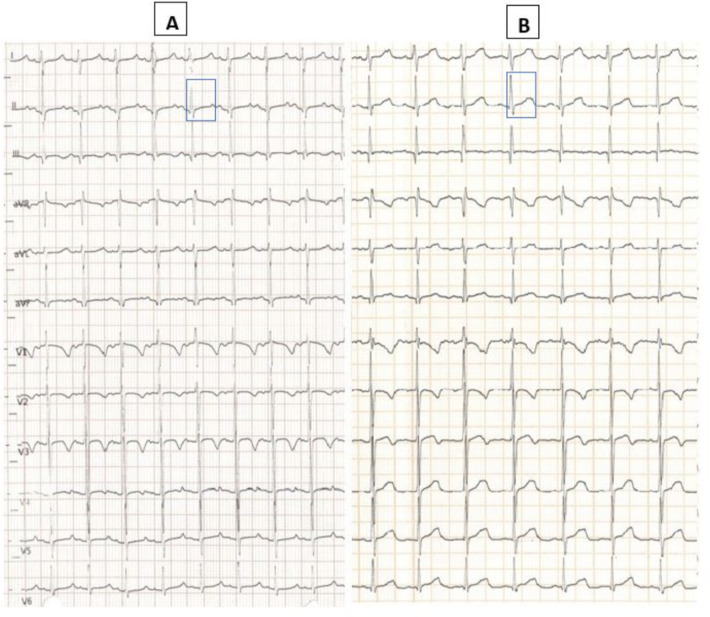
Baseline ECG at admission with QT interval corrected with Bazett formula 470 ms **(A)**; post-treatment ECG (infusion with 10% calcium gluconate) with normalized QTc interval 430 ms **(B)**.

Laboratory blood tests revealed significant hypocalcemia [6.9 mg/dl (normal values: 9–11 mg/dl)], mild hypokalemia [3.0 mEq/L (normal values: 3.5–5.5 mEq/L)], and elevated alkaline phosphatase [467 UI/L (normal values: 5–462 UI/L)]. Serum levels of sodium, magnesium, phosphate, and albumin were within normal limits. Complete blood count, renal function tests, liver and pancreatic function tests, ammonia, creatine kinase, and C-reactive protein were also unremarkable. Arterial blood gas analysis confirmed a low ionized calcium level [0.81 mmol/L (normal values: 1.12–1.32 mmol/L)] and revealed alkaline PH [7.64 (normal values: 7.35–7.45)] with mild hypocapnia [27 mmhg (normal values: 35–54 mmhg)], hyperbicarbonatemia [31.5 mmol/L (normal values: 22–26 mmol/L)] and mild hypochlorhydria [96 mmol/L (normal values 98–106 mmol/L)]. Electrocardiogram showed a prolonged corrected QT interval (QTc) with Bazett formula: 470 milliseconds (ms) (Figure 1A).

Given the hypocalcemia and the mild hypokalemia found in association with QT interval prolongation, we immediately administered 10% calcium gluconate and potassium supplements intravenously, and the child was kept nil by mouth.

We observed a progressive increase in the blood levels of these electrolytes and normalization of QTc values according to Bazett (430 ms, Figure 1B) within 24 h after the correction. Once we ruled out other mechanical causes of vomiting with an abdominal x-ray with contrast medium, the child started oral feeding again and we observed that the episodes of postprandial vomiting ended with the rapid resumption of feeding with good tolerance. Nevertheless, we performed complete diagnostic work up to exclude possible mechanical and infectious causes of vomiting, including an abdominal ultrasound, and microbiological tests on the blood and stools which came back normal.

**Figure 2:**
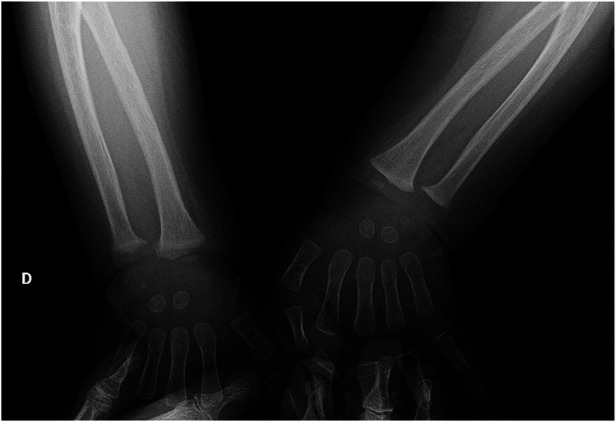
Wrist radiographs show cortical irregularities at the distal radio-ulnar metaphyseal level, prevalent in the right (D) wrist with enlargement of the growth cartilage.

Severe hypocalcemia and the history of recent vitamin supplementation withdrawal led to the clinical suspicion of rickets, therefore we requested a complete assessment of phospho-calcium metabolism which showed significant deficiency of 25-hydroxyvitamin D (25-OH D) [less than 4 ng/ml (normal values: 20–50 ng/ml)] with increased PTH [254 pg/ml (normal values: 6.5–36.8 pg/ml)]. A spot urine calcium-to-creatinine ratio excluded hypercalciuria. The wrist radiographs confirmed the diagnostic suspect (Figure 2), as they showed cortical irregularities at the distal radio-ulnar metaphyseal level, prevalent in the right wrist with enlargement of the growth cartilage. An echocardiographic examination ruled out the presence of dilated cardiomyopathy which is a possible complication of rickets.

Given the values of hypovitaminosis and hypocalcemia, oral supplementation of vitamin D3 (cholecalciferol 2,000 IU/day) associated with calcium carbonate (500 mg/day of elemental calcium, to prevent “hungry bone” syndrome) was started.

A rapid response to the administration of regular doses of cholecalciferol allowed the diagnosis to be directed towards the nutritional form of rickets. However, blood samples were taken from the child and both parents for analysis of genes associated with rickets, which later proved negative in all family members.

The child was discharged on day 8 in the absence of symptoms and with improved blood tests and continued with oral supplementation of calcium carbonate and cholecalciferol at home. Arterial blood gas analysis before hospital discharge showed a complete normalization of acid-base homeostasis (pH: 7.35, pCO2: 39 mmHg, HCO3: 20.8 mmol/L).

At the outpatient check-up performed two weeks after discharge, the child appeared in good general clinical condition, feeding well with good tolerance and gaining weight. Control blood tests showed normalization of calcium (11.2 mg/dl), phosphate (5.4 mg/dl), alkaline phosphatase (417 IU/L), and PTH (9 pg/ml), with a significant increase in 25-OH D (39.4 ng/ml). Oral calcium supplementation was discontinued after the first follow up visit, while cholecalciferol administration continued to maintain adequate vitamin D levels. Table 1 shows the blood values of calcium-phosphate metabolism from the beginning to the last check-up.

**Table 1:** Blood values of calcium-phosphate metabolism from the beginning to the last check-up.

Investigation	Reference values	Day 1	Day 3	Day 7	Day 15
Calcium	9–11 mg/dl	6.9 mg/dl	9.3 mg/dl	9,9 mg/dl	11.2 mg/dl
Ionized calcium	1.12–1.32 mmol/L	0.81 mmol/L	1.29 mmol/L	1.38 mmol/L	
pH	7.35–7.45	7.52	7.37	7.35	
Phosphate	4.5–6.5 mg/dl	4.1 mg/dl	3.6 mg/dl	2.8 mg/dl	5.4 mg/dl
Magnesium	1.46–3.16 mg/dl	2.17 mg/dl	2.42 mg/dl	2.25 mg/dl	
Albumin	3.5–5 g/dl	4.4 g/dl		4.2 g/dl	
Alkaline phosphatase	5–462 UI/L	467 UI/L		584 UI/L	417 UI/L
Parathyroid hormone	6.5–36.8 pg/ml	254 pg/ml			9 pg/ml
25-hydroxyvitamin D	20–50 ng/ml	<4 ng/ml		10.4 ng/ml	39.4 ng/ml

## Discussion and conclusion

Nutritional rickets is currently seen in children due to an increasing number of dark-skinned immigrants, lack of sunlight and food fortification, increased use of sunscreen and other skin-protective barriers, ineffective supplementation programs, etc. (4). Our patient was dark skinned and developed a clinical picture compatible with nutritional rickets, likely related to absence of adequate vitamin supplementation and poor exposure to sunlight.

The presenting symptom for which the patient was brought to our attention was vomiting. To our knowledge, this is the first reported case of hypocalcemia-induced postprandial vomiting in a pediatric patient diagnosed with rickets. Indeed, GI symptoms such as vomiting, nausea, dysphagia and abdominal pain have already been reported as atypical symptoms of calcium disbalances in children (8) but they have predominantly been linked to hypercalcemia (9, 10).

The role of calcium at various levels of the GI tract is well-supported by several articles in the literature. Indeed, smooth muscles of the GI system have scarce stores of intracellular calcium, and their contraction is directly dependent from extracellular calcium (11). Consequently, calcium plays a role as the primary regulator of intestinal muscle contraction and the calcium-sensing receptor (CaSR) is expressed in all the organs of the GI system (12, 13). In clinical practice, normal blood calcium levels have been demonstrated essential for the correct esophageal muscle contraction and spontaneous tone of both upper and lower esophageal sphincter (LES) (11, 14); consistently, systemic administration of calcium blockers have been demonstrated to decrease LES tone and increase gastroesophageal reflux (RGE) in healthy volunteers (15). Additionally, calcium has a role in regulation of gastric acid secretion and the CaSR is confirmed to participate in the regulation of calcium-induced gastric acid secretion, by increasing the intracellular calcium level and promoting the activity of the hydrogen-potassium-adenosine triphosphatase (H+-K+-ATPase) of parietal cells as well as modulating gastrin secretion (12, 13). Intravenous administration of calcium has been associated with increased carbonic anhydrase activity in the gastric mucosa and increased gastric acid secretion; conversely, administration of calcium-binding drugs has been shown to reduce blood calcium levels, while simultaneously reducing carbonic anhydrase activity and significantly inhibiting gastric acid secretion (16). Disbalances in calcium levels have been described in adults with dysphagia (17) and intractable vomiting requiring percutaneous endoscopic gastrostomy placement (18).

Taking all this evidence into account, we can assume that hypocalcemia altered the normal contractility of the esophageal smooth muscle and reduced the normal gastric acid secretion, contributing to the episodes of vomiting after the meal. The exclusion of the other potential causes of vomiting and the resolution of emesis with electrolyte correction suggests hypocalcemia as the primary cause of the symptoms. Indeed, a complete laboratory and instrumental diagnostic work-up was performed that led to the exclusion of other more common causes of vomiting in pediatric age. The instrumental exams allowed to exclude mechanical obstructive causes of vomiting (e.g., pyloric stenosis, intestinal stenosis or atresia, intussusception, malrotation with volvulus, other causes of intestinal occlusion, etc.); furthermore contrast x-ray ruled out gastroesophageal reflux (GERD) and other esophageal motility disorders. Finally, blood and stool tests were negative for viral antigens and bacteria, excluding infectious or other metabolic causes of vomiting.

The resolution of vomiting following the correction of hypocalcemia, urgently needed to reduce the arrhythmic risk resulting from QTc prolongation, constituted an ex juvantibus criterion for the causal link. The metabolic alkalosis observed at admission and related to the multiple episodes of vomiting could have contributed to lowering the ionized calcium levels and establishing a vicious circle with further alteration of gastric-esophageal motility and hypochlorhydria. Indeed, our patient presented to ED with alkalosis which probably further lowered the ionized calcium levels and worsened the effect of a latent hypocalcemia which was later proved by measuring the total serum levels.

**Figure 3:**
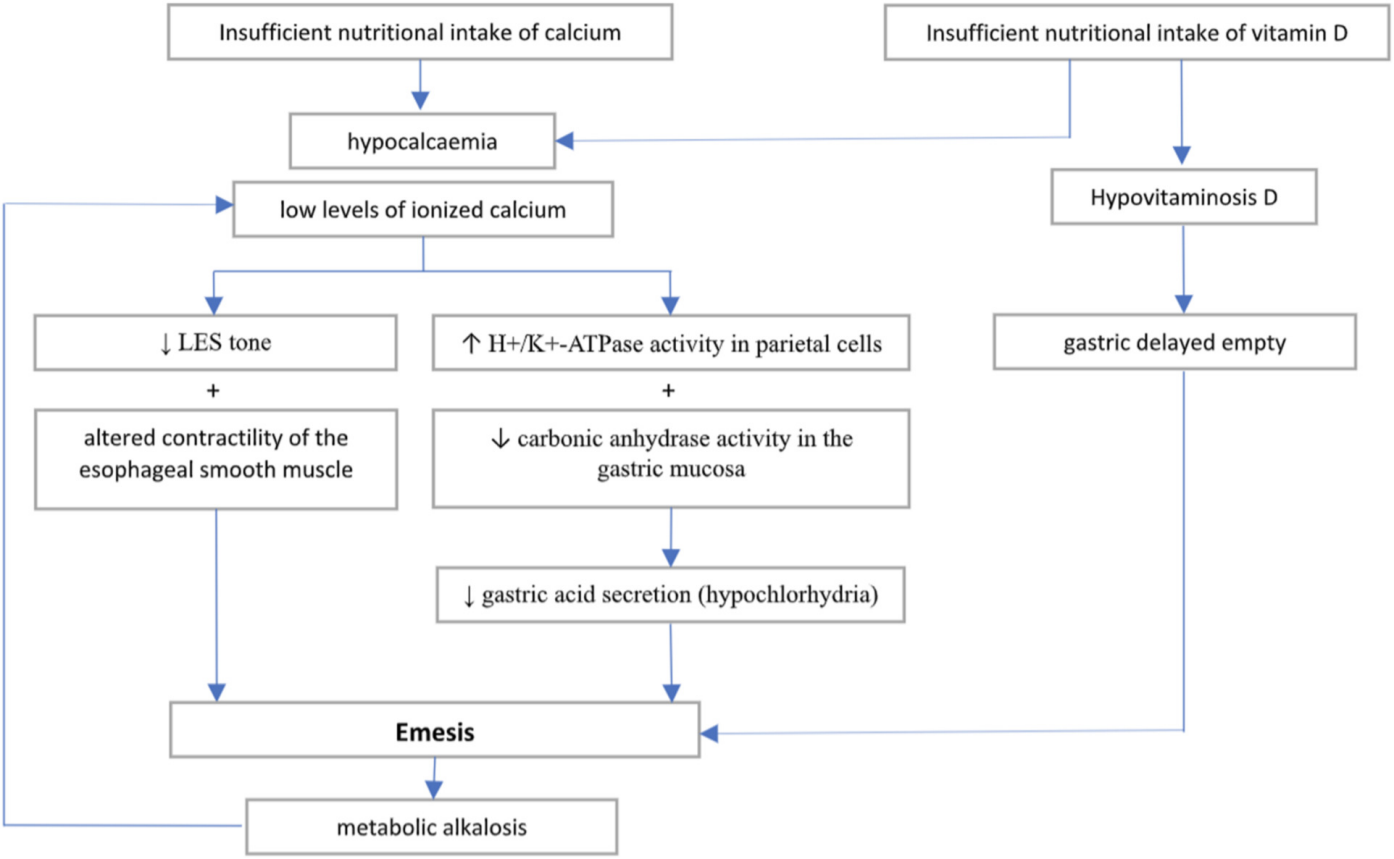
Hypothesised pathogenic mechanism causing emesis in hypocalcemia and hypovitaminosis D.

Furthermore, low levels of vitamin D have been described in patients with gastroparesis and suggest a potential role of 25-OH D in modulating gastric motility (19). Indeed, vitamin D supplementation may result in improvement of gastric delayed empty (20). Therefore, we may speculate that the effect of electrolyte disbalance in our patient may have been worsened by a nutritional deficiency of vitamin D. Figure 3 outlines the possible pathophysiological mechanisms involved in the onset of hypocalcemia-related vomiting.

The importance of detection and subsequent correction of calcium disbalances lies in the life-threatening consequences of hypo- or hypercalcemia. Specifically, hypocalcemia may lead to impairment of cardiac function in terms of both altered myocardial contraction and prolonged QT interval with consequent increased arrhythmic risk (21). Our patient experienced a prolongation of the QT interval which promptly normalized after hypocalcemia correction. Furthermore, low levels of calcium have been associated with neurological complications which have been observed in conditions of severe calcium disbalances (22).

Being the first case reported of hypocalcemia-related vomiting in a child, we would like to raise the awareness of clinicians on this symptom as a possible atypical presentation of rickets in absence of other classical features in children with suggestive nutritional or family history.

## Data Availability

The original contributions presented in the study are included in the article/Supplementary Material, further inquiries can be directed to the corresponding author.
